# Dental Legislation

**Published:** 1895-01

**Authors:** R. B. Weiser


AMERICAN JOURNAL

—OF—

DENTAL SCIENCE.

VOL. XXVIII. BALTIMORE, JANUARY, 1895. NO. 9.

ARTICLE I.

### DENTAL LEGISLATION.

BY DR. R. B. WEISER.

The object of my paper, "Dental Legislation," is one that will interest you all, I hope, and is of great importance: I give you in this paper a brief history of all the dental laws of this country, and many of the dental laws of foreign countries, condensed from all the works that I have been able to find upon the subject of dental legislation. My object in writing this paper is to call attention to the different dental laws of this country and foreign countries, so that, if any amendments to the present dental law of Colorado should be thought advisable, we may clearly understand what we are doing, and use these laws for our guide. With that object in view, I have given a very brief review of all the dental laws of this and many foreign countries, with comments upon the good points and a few suggestions that I deem of importance.

Beginning with Alabama, we find that Alabama has a dental law that requires a board of examiners of five, elected by the State association; diplomas or certificates of other States are not recognized. All who desire to practice in this State must go before the board and be examined. The fee is $5; *provided*, that dentists who have been in regular practice for five years next preceding the passage of this act shall not be required to pass an examination and shall be entitled to a license without fee. Any member of the board may grant a temporary permit. The board must prescribe a course of study for those who desire to study dentistry under private instructions; all certificates must, within thirty days, be presented to the judge of the probate court of the respective county, who shall officially indorse and seal it with the seal of the court, and shall record said license; the fee for which is $1. Under the first law of Alabama, the board of health, with the addition of one dentist, examined all applicants. The law of Alabama was the first dental law in this country, and, in my opinion, the first genuine dental law in the world. England had a law that had some reference to dentistry—or rather to the extraction of teeth—enacted in the thirty-second year of the reign of Henry VIII. By the 3d section of this act, any one who uses "barbery or shaving" in the city of London—its suburbs, and within a circuit of one mile—is forbidden occupying any surgery, letting of blood, or any other thing belonging to surgery, drawing of teeth only excepted. (Henry VIII was born in 1491; he began his reign when he was 18 years old, which would be in 1509; consequently the thirty-second year of his reign would be 1541,) I am still of the opinion that the dental law of Alabama was the first genuine dental law in the world. This law was passed in 1841; it was amended in 1881; and again amended in 1887.

Arkansas has a law which requires a board of examiners of five, appointed by the Governor; diplomas are recognized. The board simply pass upon the validity of the same. All who were engaged in the practice at the time of passage of this act are admitted without examination, the fee for which is $1; all other examinations $5. The law is not very clear—in fact, it is almost impossible to determine what it means; but I am of the opinion that none but graduates are admitted now. This law was passed in 1887.

Arizona has no dental law (unless one has been passed recently, within the last two years.)

California has a law which requires a board of examiners of seven, appointed by the Governor. Any and all persons who shall so desire may appear before said board; said board shall also indorse as satisfactory diplomas from any reputable dental college. All dentists in the State at the time of the passage of the law were admitted to practice without examination upon the payment of a fee of $1; the examination fee is $10. All certificates must be registered with the county clerk, the fee for which is $1. All fines collected go to the school fund. This law was passed in 1887.

Colorado has a law requiring a board of dental examiners, five in number, appointed by the Governor. The time and place of meeting is optional with the board. All who apply for license must either have a diploma from a reputable dental college or a certificate from the board of dental examiners of some other State. Neither a diploma or certificate will exempt the holder from an examination, the examination fee is $10. Temporary permits issued by the Secretary. Approved May 15, 1889, and amended April 1, 1891.

Connecticut has a so-called dental law; no board, no examination, no fees. This law was passed in April, 1887, and has never been amended. Diplomas or certificates will admit to practice without examination.

Delaware has a law requiring a board of examiners, five in number, appointed by the Governor; diplomas are recognized; no fee for examination; $1 for the certificate; *any and all* persons may go before the board. Passed March 31, 1885, and has never been amended.

The District of Columbia has no dental law.*

Florida has a dental law, and a good one, requiring a board of examiners, five in number, appointed by the Governor. Only those having diplomas are allowed to practice, but diplomas do not exempt the holder from examination. The duty of the board is to grant certificates to all applicants who have obtained a diploma from a reputable dental college, and who pass a *satisfactory examination*. The fee is $10. This law was passed in 1887 and amended in 1891.

Georgia has a law which requires a board of examiners of five, selected by the State association. Diplomas are not recognized; any and all persons may go before the board; any member of the board may issue a temporary permit, all must register with the clerk of the Superior Court; the fee for this is fifty cents; there is no fee for examination. One-half of all fines collected go to the informer, the other half to the school fund. The law was passed in 1872, amended in 1875, and again amended in 1879.

Georgia has another important law to which I desire to call attention, under the title of "An act to exempt all regular practitioners of dentistry from jury duty."

"SECTION 1. That from and after the passage of this act all dentists who are in regular practice in this State shall be exempt from all jury duty.

"SEC. 2. That all laws and parts of laws in conflict with this act are hereby repealed."

This law was passed in 1881, and, so far as I can learn, is the only law of the kind in this country.

Indiana has a law requiring a board of dental examiners, who are appointed as follows: The State dental association elects three, the Governor appoints one, and the State board of health appoints one; diplomas are recognized; the examination fee is $5. Passed in March, 1879.

---

*The District of Columbia has a dental law.

Iowa requires a board of dental examiners, five in number, appointed by the Governor; diplomas are recognized; the fee is $2; any and all persons may go before the board. Approved March 2, 1882.

Illinois requires a board of examiners, consisting of five, appointed by the Governor; diplomas are recognized; medical diplomas will admit the holder to examination; the fee for certificates for graduates (without examination) is $1; all others $2. By this law the fees of the board of examiners is fixed at $5 a day, with traveling expenses. I am unable to find the date of the passage of this law.

Kansas has a law and requires a board of dental examiners, consisting of four, appointed by the Governor; none but graduates of reputable dental colleges are admitted; certificates of qualification from other State boards of dental examiners are not recognized; applicants must have a diploma from some dental college, provided that nothing in this act shall apply to any person engaged in the practice of dentistry in this State at the time of the passage of this act, and provided, further, that nothing in this act shall be so construed as to prevent physicians, surgeons and others from extracting teeth; for certificates to those in practice at the time of the passage of this act the fee is $3, to all others $10; the board receive $5 a day and all legitimate expenses. This law was passed in 1885.

Kentucky requires a board of examiners, consisting of five, elected by the State association. The president and secretary of the State dental association are elected to their respective offices and as members of the board of dental examiners at the same time; three additional examiners are then elected by the association. The State associations control everything, and all certificates are issued in their name; diplomas are recognized; the fee is $20; all money is turned over to the State association, and reports are made to the same; the State has nothing whatever to do with it; this association is incorporated. Violators of this law are indicted by the grand jury of the county in which the offense was committed, and all fines collected go to the school fund; the remuneration of the board is also controlled by the State association. Passed in 1868.

Louisiana requires a board of dental examiners of five, elected by the State association; diplomas are recognized; two members of the board may grant a temporary permit; violators are indicted by the grand jury of the county in which the offense was committed; of the fines collected one-half go to the State dental society and the other half to the school fund; no fee whatever. This law was passed in 1880.

Maine requires a dental board of five, appointed by the Governor; diplomas are not recognized; any and all persons may go before the board; all must pass an examination in anatomy, physiology, pathology, therapeutics, chemistry and the thecry and practice of dentistry; the secretary may grant temporary permits; the fee is $20. Enacted in 1891.

Maryland requires a board of examiners of five, appointed by the Governor; diplomas are recognized, and holders of the same do not have to pass an examination; any and all persons may go before the board; any member of the board may issue a temporary permit; the fee is $1; and fines collected go to the school fund. This law was passed in 1884 and amended in 1886.

Massachusetts requires a board of examiners of five, appointed by the Governor; diplomas are not recognized; any and all persons may go before the board; the fee is $10; the examinations are either oral or written, at the option of the applicant; the board receive five dollars a day and three cents a mile each way. This law was passed in 1887.

Michigan requires a board of examiners of three, appointed by the Governor; diplomas are recognized; any member of the board may issue a temporary permit, and may also appoint a substitute to act in his absence; the fee is $13, three dollars for the temporary permit and ten dollars for the certificate. This law was enacted in 1883.

Minnesota's dental law requires a board of examiners of five, appointed by the Governor from ten persons selected by the State dental association. To be eligible for examination, the applicant must have a diploma or ten consecutive years of actual practice. The fee is $10. This law was passed in 1885 and amended in 1889 The amendment is very elaborate and contains much very important legislation. The examinations are written and are provided for by law as follows: Anatomy, physiology, chemistry, materia medica, therapeutics, metallurgy, histology, pathology, operative and surgical dentistry, mechanical dentistry and demonstrations of their skill in operative and mechanical dentistry. Every dentist practicing in this State must register every year, the fee for which is $1. The board is incorporated. All fines collected go to the school fund; justices of the peace and the respective municipal courts shall have jurisdiction over violations of this act. This law has been carried to the Supreme Court and has stood the test.

Mississippi requires a board of dental examiners of five, appointed by the Governor; diplomas are recognized; the fee for graduates is $2, others $5; certificates must be recorded; the minimum fine is $10, unlimited the other way. This law was passed in 1882 and amended in 1890. The amendment does not recognize diplomas.

Missouri has a dental law, but no board of examiners. None but graduates of dental colleges are permitted to practice, except those in the State at the time of the passage of this law; the diploma or a certified copy must be filed with the clerk of the county court, or, if in St. Louis, with the city register; a certificate is issued by the clerk or register and recorded; the fee is $1. This law was passed in 1883.

Montana has no dental law, unless one has been enacted recently within the last two years.

Nebraska has a dental law without a board of examiners; none but graduates of dental colleges can practice in this State, except those in the State at the time of the passage of the law; the diplomas must be presented to the county clerk, who shall file and register the name of the person, the date of filing, and the name of the college; the fee is $1. The dental law of Nebraska is exactly the same as the dental law of Missouri enacted in 1887.

New Hampshire requires a board of censors, consisting of three elected by the State dental society; diplomas are recognized; the fee is $5; passed in 1885, amended in 1887. By the amendment, diplomas are recognized, but all must register, and the board of censors is changed to the board of registration in dentistry, appointed by the Governor.

New Jersey requires a board of examiners of five, elected by the State association; diplomas are recognized; the fee is $30; one-half of all fines collected go to the informer, the other half to the school fund; any and all persons may go before the board. The law was passed in 1873, amended in 1880 and again amended in 1882. In 1890 an entire new law was enacted. By the new law the board of examiners is changed to the board of registration and examination in dentistry. The State dental society shall recommend to the Governor five dentists of good repute whom the Governor shall appoint, and no person shall be examined by said board unless he is a graduate or the holder of a diploma of graduation from a dental college recognized as in good repute by said board, or shall have studied with a reputable dentist for five years continuously in this State. The fee is reduced to $25, and half of all fines collected go to the board, the other half to the State. Approved April 7, 1890.

New York has a very peculiar and elaborate dental law, differing from all others in many respects. They first organize eight district dental societies corresponding with the eight judicial districts of the Supreme Court. Each of these district dental societies appoints a board of censors of three or five, called the district board of censors.

Each of the eight district societies elects annually eight delegates (who at the first meeting organize a State dental society), and who are known as delegates to the State dental society. The State dental society elects eight censors —one from each district society—who constitute the State board of censors. The board of censors of the several districts examine all persons who present themselves for examination, and report, in writing, to the president of the district society, who shall thereupon issue, on the recommendation of said board of censors, a certificate of qualification to such person, countersigned by the secretary and bearing the seal of said district society. The fee for this is $10. The State board of censors examine all who hold district certificates and report their opinion, in writing, to the president of the State dental society, who, upon the recommendation of said board, shall issue to such person or persons a diploma conferring upon him the degree of Master of Dental Surgery—M. D. S. The fee for this is $20; this degree is optional; a certificate from the president of any one of the eight district societies will permit any person to practice dentistry in New York; all must register; the fee for which is fifty cents; diplomas of reputable dental or medical colleges are recognized; the State society have the power to determine as to the reputability of the college. The dental law of New York was enacted in 1868 and has been amended three times, making four enactments, the last of which was in 1888; each of the district societies and the State society are incorporated.

North Carolina has a board of examiners of six, elected by the State association; diplomas were recognized by the original act, but are not recognized by the amendment; all must pass an examination except regularly authorized physicians and surgeons. Every certificate must bear the seal of the State society; there is no fee whatever; any member of the board may issue a temporary permit. This law was passed in 1879 and amended in 1883.

North and South Dakota require boards of examiners of five appointed by the respective Governors. Diplomas are recognized, but all must pay the examination fee, which is $10, and an additional registration fee of $5. This registration certificate entitles the holder to registration, which he must do annually, the fee for which (after the first year) is $2; the fees are, therefore, $15 for the first year and $2 a year thereafter. Any member of the board may issue temporary permits. This law was passed in 1890.

Nevada has no dental law.

Ohio has a dental law, but no board of examiners. All who practice dentistry must have a diploma or certificate of qualification from the State dental society. All prosecutions are by indictment before the court of common pleas in the county wherein the offense was committed, and all fines collected go to the school fund; there is no fee. This law was passed in 1868 and amended in 1873.

Oregon requires a board of examiners, appointed by the Governor; diplomas are recognized and holders of the same are granted certificates without examination upon payment of a fee of $5; all others $10. All must go before the board.

Oklahoma requires a board of examiners of five, appointed by the Governor; diplomas are recognized. All must go before the board and pay the fine of $10, except those engaged in the practice three months before the passage of this act, in which case the fee is $3. Holders of dental diplomas are not examined. This law was passed in 1890.

Pennsylvania requires a board of examiners of six, elected by the State dental association. Nothing but a dental diploma will admit to practice in this State, provided that nothing in this act shall apply to persons who shall have been engaged in the continuous practice of dentistry in this State for three years at the time of the passage of this act. The fee under the original act was $30; by the amendment there is no fee. Certificates from other State boards are not recognized. All prosecution is by indictment and all fines collected go to the poor fund. This law was passed in 1876 and amended in 1883.

Rhode Island requires a board of registration of five, appointed by the Governor. All persons not graduates of dental colleges who may desire to practice dentistry in this State, subsequent to the passage of this act, may appear before said board, and to such as undergo a satisfactory examination, certificates to that effect shall be issued. After the first three months after the passage of this act, none but graduates of dental colleges are admitted; the fees are $27—$2 for examination and $25 for the certificate; graduates are not examined, but must pay the fee. This law was passed in 1888.

South Carolina has a board of examiners of five, elected by the State association. Diplomas are recognized and holders of the same are admitted without fee, charge or examination; all others must pass a satisfactory examination, the fee for which is $15; all must become members of the State association. Every dentist in the State must keep a record of each and every case in duplicate and furnish a copy to the patient if so desired; all prosecutions are by indictment before the grand jury; all fines collected go to the school fund. This law was passed in 1875.

Tennessee requires a board of examiners consisting of six, two from each of the three subdivisions of the State—East, Middle and West—Tennessee, appointed by the Governor; diplomas are recognized; all others must pass a satisfactory examination; the fee is $5. This law was passed in 1891.

Texas requires a board of examiners of three, appointed by the judges of three judicial districts; diplomas are recognized but all must go before the board; the fee is $5; any member of the board may grant a temporary permit; all fines collected go to the school fund; all certificates must be recorded in the respective counties. This law was passed in 1889.

Vermont requires a board of examiners of five, appointed by the Governor; diplomas are recognized and no fee or examination from holders of the same is required; for others the fee is $5; all certificates must be recorded; any member of the board may grant a temporary permit; the board receives $3 per day and no mileage. This act was passed in 1882.

Virginia requires a board of examiners, consisting of six, appointed by the Governor from twelve candidates named by the State association; diplomas are recognized; all others must be examined; the fee is $10; all fines collected go to the school fund. This law was passed in 1886.

West Virginia requires a board of examiners of nine, appointed by the board of public works, three from each congressional district; diplomas are recognized; all others must be examined, the fee for which is $18, $2 to each member of the board. Approved February, 1881. This law was amended in 1883. By the amendment the board consists of twelve, appointed as aforesaid; each member of the board is entitled to a fee ot $2, paid by the applicant; the examination fee, therefore, by the amendment, is $24. Passed in 1883.

Wisconsin requires a board of examiners of five, appointed by the Governor; diplomas are recognized; all must go before the board; graduates are not examined; the fee for them is $1, for others $10. The law was passed in 1885.

Washington requires a board of examiners of five, appointed by the Governor; diplomas are recognized; all must go before the board, but no examination for graduates; the fee is $25 for all classes; all certificates must be registered; two members of the board may issue a temporary permit. The act was passed in 1888.

Wyoming has a dental law, without a board of examiners. None but graduates of dental colleges can practice dentistry in this State; provided that nothing in this act shall apply to any *bona fide* practitioner of dentistry in the State at the time of the passage of this act. A copy of his or her diploma must be filed with the county clerk of the county, under oath. The clerk of the county shall give a certificate, with the seal of the county attached; no fee whatever, unless to the county clerk.

In addition to the laws of this country we find that almost all civilized nations of the world have some dental legislation. Some of the laws of foreign countries are very elaborate indeed, and cover page after page of printed matter. We regret that time and space will not permit us to review these laws, but such is the case, and we can, therefore, only give the name of the country in which dental laws have been enacted with a brief reference to one or two. They are Austria, Hungary, Belgium, Brazil, Cuba, Denmark and Danish provinces, England, France, Germany, Dominion of Canada, British Columbia, Manitoba, New Brunswick, New Zealand, Victoria, Nova Scotia, Ontario, Prince Edward Island, Quebec, Holland, Italy, Mexico, Russia and Spain. Nearly all of these countries require a board of dental examiners appointed by the crown or boards of health or elected by the dental associations. In many countries dental diplomas are recognized, and in others they are not. A fee of some kind is usually charged. In many places the examinations are very rigid indeed, and are prescribed by law.

The dental laws of all foreign countries are very similar to the American laws, from which many of them seem to have been copied:

Italy provides that no person shall practice dentistry unless holding the degree of medicine and surgery.

In New Zealand the law requires registration, and the first qualification for registration is that they should show that they are of good moral character.

In Denmark any person desirous of practing dentistry must first pass an examination in Danish history, geography, geometry, algebra, English or French. The examinations are conducted by members of the medical faculty and several dentists appointed by the board of health. In mechanical dentistry the candidate must give proof of his skill by the "setting and insertion" of a single artificial tooth and six partial or full dentures. In operative dentistry he must also, by practical demonstration, prove his ability to perform the various operations in the mouth. He must procure the patients and materials himself, the work being done in the laboratory of some one dentist of the board, who will have special control over these tests. The work must be done within fourteen days, and be judged by the whole board. The dentist appointed as special supervisor for this work receives 20 riss (a riss is 50 cents), $10. The theoretical trial or examination is conducted by the whole board, verbally, and is as follows: Anatomy and physiology of the face, teeth, gums and mouth; pathology of the mouth, teeth, gums and jaw bones, also diseases of the teeth and gums; the preparation and effect of medicine in dentistry; a knowledge of dental instruments and their application; indications for the different operations and their practical execution. The examination fee is 20 riss, $10. No dentist is allowed to administer an anæsthetic without the presence of a medical practitioner.

France the same.

In conclusion, Mr. President, I wish to call attention briefly to the dental law of Colorado. You are all familiar with the details of this law, so that it will not be necessary for me to go into the history of it any further than to say that dental legislation was attempted in the fifth General Assembly, but unfortunately it was defeated. It was again attempted in the sixth General Assembly and again defeated. In the seventh General Assembly we were more fortunate, and succeeded in passing Colorado's first dental law. This law, after being in operation for two years, was amended, as you all know, in the eighth General Assembly, or in 1891. The dental law of Colorado has been very severely criticized, and some have condemned it entirely.

Notwithstanding the fact that it has been so severely criticised, I am firmly of the opinion that it will compare very favorably with any dental law in this country, and that it will stand the test of the Supreme Court. I admit frankly that it has some slight defects, and that possibly it should be amended. I have read with much care the dental law of every State in this country, and many of the dental laws of foreign countries, and find that all of them have some defects, with possibly one single exception, namely, Florida. It would seem, Mr. President, that with all this data before us it would be an easy matter to formulate a dental law that would be absolutely perfect, but such is not the case. It is not an easy matter; on the contrary, it is an exceedingly difficult matter.

Proceeding upon the hypothesis that a new dental law has been contemplated, I wish to call attention, with a view of opening a discussion upon them, to several important and prominent features of dental legislation. First of all, I believe the present law of Colorado can be converted into a perfect dental law by amendment, and I know by experience that it is easier and safer to amend an old law than to enact a new one. If anything at all is done, it should be by amendment. Save the old law if nothing is left but the enacting clause. Certificates of qualification from other State boards of examiners should not be recognized under any circumstances, for several important reasons, chief of which is the act that many of these certificatee have been issued, not upon merit, but simply upon the fact that the holder was a resident of the State at the time of the passage of the law. Every certificate in the State should be recorded in the respective counties. All credentials presented to the board should be under oath, with a heavy penalty attached for false representations. The board should have the power to determine as to the reputability of dental colleges of this and foreign countries, and should have for their guide some specific legislation. A record book should be provided for by law, and all certificates issued should be recorded in said book, with the date, name of applicant, name of dental college, State, etc. Temporary permits should be worded very carefully by statute and issued only to those making the proper affidavit. Every single thing desired or contemplated in a dental law must be set forth in plain, unmistakable Anglo-Saxon, in terms so plain that it would be a physical impossibility to put any other construction upon it. All amendments contemplated should be done under the auspices of the State association, and to that end, Mr. President, I would suggest that a committee of five, or seven, or nine, be appointed to draft any and all amendments deemed necessary, and submit the same to a special meeting of this association in the near future.—*Proceedings of Colorado State Dental Society.*

